# Multi-omics data reveal causal associations of cellular senescence-related genes in rheumatoid arthritis: A summary-data-based Mendelian randomization and co-localization analysis

**DOI:** 10.1097/MD.0000000000047376

**Published:** 2026-01-30

**Authors:** Ping Jiang, Youji Jia, Juhua Zhang, Zhi Wang, Honghong Ma, Yajuan Guo, Mingcong Wang, Wei Yan, Xiaobing Xi

**Affiliations:** a Ruijin Hospital Affiliated to Shanghai Jiao Tong University School of Medicine, Shanghai, China; b Shanghai Ruijin Rehabilitation Hospital, Shanghai, China; c Zhoupu Hospital Affiliated to Shanghai Health College, Shanghai, China; d Shanghai University of Traditional Chinese Medicine, Shanghai, China.

**Keywords:** cell senescence, gene expression, Mendelian randomization, methylation, protein, rheumatoid arthritis

## Abstract

Rheumatoid arthritis (RA) is a complex autoimmune disease. Recently, cell senescence has been identified as a key factor in its pathogenesis. This study integrated multi-omics summary data and applied Mendelian randomization (MR) and co-localization analysis to systematically evaluate the causal relationships between cell senescence-related genes and RA. We collected summary data on blood methylation quantitative trait loci (mQTL), expression quantitative trait loci, and protein quantitative trait loci. The FinnGen database was the primary discovery dataset, validated by the UK Biobank and GWAS Catalog. We used the summary-data-based MR method to assess causal associations between the molecular traits of cell senescence-related genes and RA. Co-localization analysis was then performed to confirm shared genetic variants. After integrating multi-omics data on cell senescence-related mQTL and expression quantitative trait loci, we identified 5 key cell senescence-related genes potentially associated with RA: *BCL2L1*, *DNMT3B*, *ERRFI1*, *NEK4*, and *RAF1*. These genes demonstrated significant causal associations across multiple analyses. The mQTL signals based on summary-data-based MR analysis show that the genetically regulated methylation variations at the cg12873919 (odds ratio [OR] = 0.91, 95% CI [0.84–0.99]) and cg13989999 (OR = 0.90, 95% CI [0.82–1.00]) sites of the *BCL2L1* gene are negatively associated with RA risk and may mediate disease risk by upregulating gene expression (OR = 0.82, 95% CI [0.76–0.88] and OR = 0.78, 95% CI [0.71–0.87]). Conversely, the mQTL effect size at the cg26432171 site of the *RAF1* (OR = 1.17, 95% CI [1.02–1.33]) is positively associated with RA risk and is consistent with the upregulation of gene expression (OR = 1.83, 95% CI [1.49–2.25]), thereby enhancing RA susceptibility. Moreover, several sites in the *DNMT3B* gene (e.g., cg09149842) exhibited negative correlations with RA risk, suggesting that *DNMT3B* may play a critical role in RA pathogenesis by affecting gene expression. Methylation sites in *ERRFI1* (cg13808198, cg22678073) and *NEK4* (cg09524078) were also associated with RA risk, supporting their potential regulatory roles in RA. Co-localization analysis further validated the association between methylation sites and RA, particularly for *BCL2L1*, *RAF1*, *DNMT3B*, *ERRFI1*, and *NEK4*, where we identified shared causal signals with RA (posterior probability of H4 > 0.5). This study systematically evaluated the causal relationships between cell senescence-related genes and RA risk. These findings provide new insights into RA pathogenesis and reinforce the clinical value of these genes as potential therapeutic targets.

## 1. Introduction

Rheumatoid arthritis (RA) is an autoimmune disease characterized by synovitis, which can ultimately lead to joint deformities. The etiology of RA remains under investigation, but studies suggest it is closely related to factors such as autoimmunity, genetics, and microbial infections.^[1,2]^ Accelerated biological aging is considered a major risk factor for age-related diseases or mortality. As senescent cells accumulate in tissues, they promote the development of age-related diseases and impact overall bodily function. Therefore, expanding our understanding of the mechanisms that control cell senescence is crucial.^[3,4]^

Cell senescence is a state of permanent proliferative arrest that cells enter after experiencing various stress factors such as DNA damage, oxidative stress, and telomere shortening. This state is accompanied by the secretion of inflammatory cytokines and matrix-degrading enzymes, a phenomenon known as the senescence-associated secretory phenotype.^[5]^ The appearance of senescence-associated secretory phenotype not only disrupts local tissue homeostasis but also exacerbates inflammatory responses by inducing senescence in neighboring cells and activating the immune system.^[6]^ The prevailing theory suggests that senescence results from the gradual shortening of telomeres during cell division, leading to DNA damage and decreased cellular function.^[7]^ During this process, the immune system may be affected, resulting in autoimmune responses, wherein normal cells are mistakenly targeted by the immune system. Such autoimmune responses may contribute to the development of autoimmune diseases like RA. Previous research indicates that accelerated cell senescence may play a role in the pathogenesis of RA. An observational cohort study conducted by Chinese researchers found that accelerated biological aging may increase the risk of RA, particularly in individuals with a high genetic predisposition, while also potentially reducing the life expectancy of RA patients.^[8]^ Related studies have shown that RA patients tend to have shorter telomeres,^[9]^ and their immune cells differ significantly in type and quantity compared to healthy individuals, exhibiting signs of accelerated senescence.^[10]^ These findings, at the molecular and cellular levels, further support the connection between RA and immune system aging, with telomere shortening-induced cellular damage being a critical factor. Other studies have also observed significant senescence characteristics in synovial cells and immune cells of RA patients. For example, fibroblast-like synoviocytes (FLS) in RA patients display reduced proliferative capacity and increased secretion of inflammatory cytokines, which are closely associated with the senescent phenotype.^[11]^ Additionally, telomere shortening and increased DNA damage have been observed in peripheral blood mononuclear cells and T cells of RA patients,^[12]^ suggesting that cell senescence may be a key factor in RA pathogenesis.

**Figure 1.**
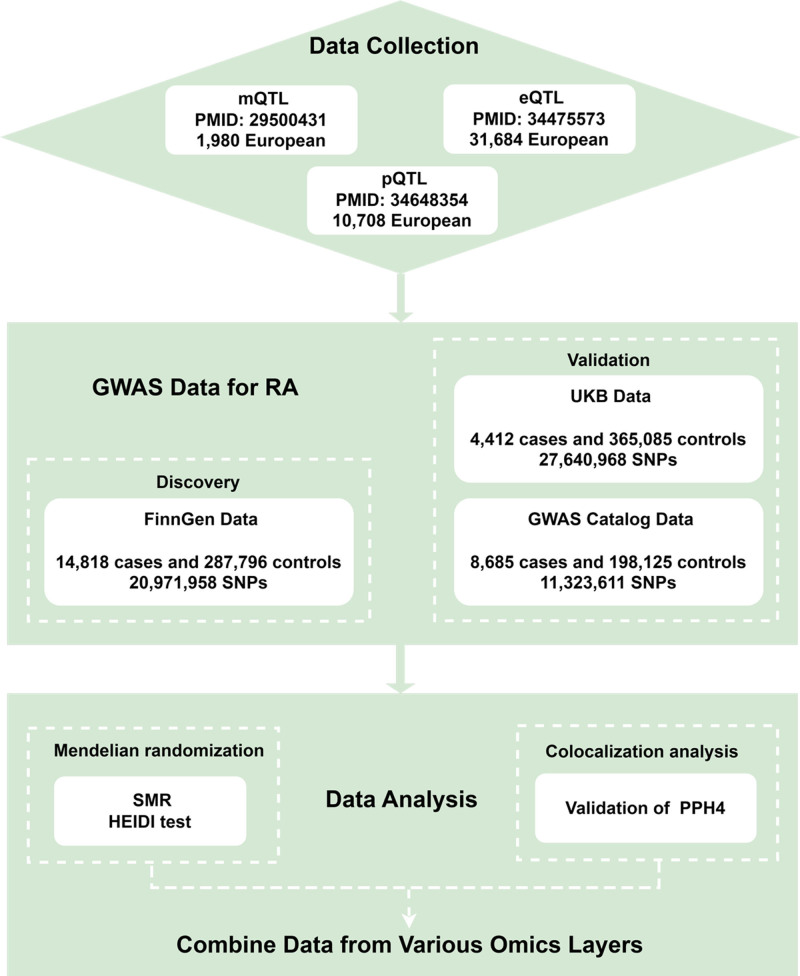
Research process. HEIDI = heterogeneity in dependent instrument; PPH4 = posterior probability of H4; QTL = quantitative trait loci; RA = rheumatoid arthritis; SNP = single nucleotide polymorphisms.

Therefore, this study aims to systematically assess the role of cell senescence in RA by integrating genome-wide association data and cell senescence-related gene expression data. We will utilize summary-data-based Mendelian randomization (SMR) and co-localization analyses to further clarify the causal relationships between cell senescence-related molecular traits and RA (Fig. 1). Through this research, we hope to uncover the mechanisms of cell senescence in RA pathogenesis, providing new potential targets and strategies for the precise treatment of RA.

## 2. Materials and methods

### 2.1. Data sources

The GWAS summary data used in this study were downloaded and accessed from the respective public databases or consortia websites between August 2024 and October 2024. Cell senescence-related genes were extracted from the CellAge database (https://genomics.senescence.info/cells/), comprising a total of 949 genes. The GWAS data for RA were obtained from multiple international datasets, including the FinnGen dataset (used for the discovery phase), the UK Biobank (UKB) dataset (used for validation), and the GWAS Catalog dataset (used for validation). Quantitative trait locus (QTL) analyses were employed to reveal the relationships between single nucleotide polymorphisms (SNPs) and DNA methylation, gene expression, and protein abundance. The methylation quantitative trait loci (mQTL) data were derived from a meta-analysis of 2 European cohorts,^[13]^ specifically the Brisbane Systems Genetics Study (n = 614) and the Lothian Birth Cohorts (n = 1366). Blood expression quantitative trait loci (eQTL) data were obtained from the eQTLGen database,^[14]^ which includes genetic data on blood gene expression from 31,684 individuals. The genetic association data at the protein level were sourced from a protein quantitative trait loci (pQTL) study conducted by Pietzner et al,^[15]^ involving 10,708 European individuals. By integrating MR analysis results from these 3 distinct levels, we identified candidate genes with potential causal relationships. Additionally, there was no sample overlap between the exposure and outcome datasets. This study exclusively used publicly available summary-level data. The authors did not have access to any information that could identify individual participants at any stage of the research.

### 2.2. Summary-data-based MR analysis

Prior to SMR analysis, we 1st performed quality control of the instrumental variables (SNPs). In the data preprocessing stage, we utilized the allele frequency of the SNPs (e.g., minor allele frequency > 0.01) as one of the quality control criteria and ensured allele consistency between the GWAS and QTL datasets. In this study, we conducted SMR analysis using the SMR (v1.3.1) tool and performed heterogeneity in dependent instrument (HEIDI) testing to assess the associations between cell senescence-related gene expression, methylation, and protein abundance with RA. By screening for the most relevant cis-QTLs within ± 1000 kb upstream and downstream of the target gene with a *P*-value <5.0 × 10^−8^, and ensuring independence between exposure and outcome data, we validated the advantages of the SMR method over traditional MR analysis. To enhance precision, we excluded SNPs with an allele frequency difference >0.2. For mQTL, eQTL, and pQTL, the maximum allowable proportion of SNPs with allele frequency differences was set at 0.05. We then used mQTL as exposure and eQTL as the outcome to explore the causal relationship between mQTL and eQTL. Similarly, eQTL was used as exposure and pQTL as the outcome to investigate the causal relationship between eQTL and pQTL. Moreover, we employed multi-SNP SMR analysis (SMR multi) combined with HEIDI testing to ensure that the results included in the analysis were significant and not confounded by pleiotropy (*P* SMR < .05, *P* SMR multi < .05, and *P* HEIDI > .05).

### 2.3. Co-localization analysis

We performed co-localization analysis using the R package coloc to identify shared causal variants between cell senescence gene-related cis-QTLs (including mQTL, eQTL, and pQTL) and RA. In the co-localization analysis, we reported the posterior probabilities corresponding to 5 mutually exclusive hypotheses: (H0) no genetic association between any trait and SNP in the region; (H1) only trait 1 is associated with SNPs; (H2) only trait 2 is associated with SNPs; (H3) both traits are associated with SNPs but are driven by different causal variants; (H4) both traits are associated with SNPs and share a common causal variant. For mQTL–GWAS, eQTL–GWAS, and pQTL–GWAS co-localization analyses, the region window was set to ±1000 kb. To ensure co-localization of QTL signals with relatively weak *P*-values, we set *P*12 = 5 × 10^−5^ and considered QTL and GWAS signals to be successfully co-localized under the condition of posterior probability of H4 (PPH4) > 0.5.

### 2.4. Statistical analysis

All statistical analyses were conducted using R software (v4.4.1). Manhattan plots were generated using the “ggplot2” and “ggrepel” R packages, while forest plots were created with the “forestplot” R package. The code for generating SMRLocusPlot and SMREffectPlot was based on the study by Zhu et al.^[16]^

## 3. Results

### 3.1. Integration of RA and blood mQTL data related to cell senescence

**Figure 2.**
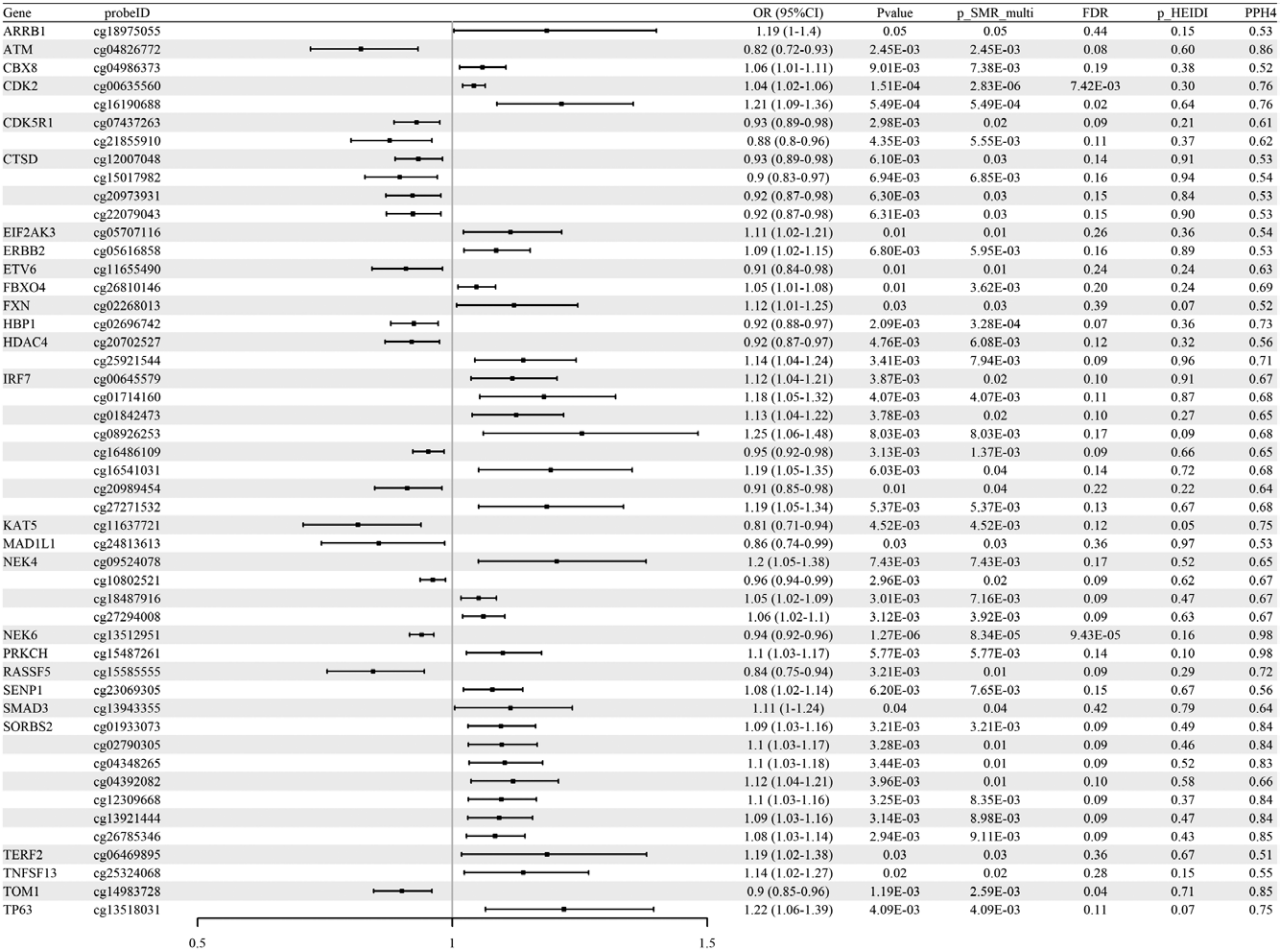
Association between cell senescence-related mQTL and RA–GWAS in SMR analysis (Forest map). CI = confidence interval, mQTL = methylation quantitative trait loci, OR = odds ratio, RA = rheumatoid arthritis, SMR = summary-data-based Mendelian randomization, SNP = single nucleotide polymorphisms.

SMR analysis was conducted to assess the association between cell senescence-related mQTL and RA–GWAS. A total of 194 CpG sites (corresponding to 92 genes) were identified as significantly associated with RA (*P* SMR < .05 & *P* SMR multi < .05 & *P* HEIDI > .05). Due to the large number of sites, which makes full presentation challenging, we have showcased 49 key CpG sites with PPH4 > 0.5 (Fig. 2), with the complete analysis results available in Table S1, Supplemental Digital Content, https://links.lww.com/MD/R241. The association between the mQTL effects of these CpG sites and RA risk was evaluated through SMR analysis, with HEIDI testing used to rule out pleiotropy. For instance, the methylation level of CpG site cg20702527 in the *HDAC4* gene was negatively correlated with RA risk (odds ratio [OR] = 0.92, 95% CI [0.87–0.97]). However, different CpG sites within the same gene did not always show consistent effect estimates. For example, another CpG site in *HDAC4*, cg25921544, was positively correlated with RA risk (OR = 1.14, 95% CI [1.04–1.24]). Subsequent co-localization analysis revealed that 49 sites (corresponding to 27 genes) among the identified results had strong evidence of co-localization within the locus region of the corresponding SNPs (PPH4 > 0.5, Fig. 2), including *ATM* (cg04826772), *CDK2* (cg00635560, cg16190688), *HBP1* (cg02696742), *HDAC4* (cg20702527), and *KAT5* (cg11637721). The initial set of 194 CpG sites was subsequently validated in the UKB and GWAS Catalog cohorts. The results showed that 7 methylation sites, including *CDK2* (cg00635560, cg16190688) and *FBXO31* (cg27365499), were validated in the UKB dataset (*P* SMR < .05 & *P* SMR multi < .05 & *P* HEIDI > .05, Table 1), with the full validation results available in Table S2, Supplemental Digital Content, https://links.lww.com/MD/R241. In the GWAS Catalog cohort, 13 methylation sites, including *ARPC1B* (cg05715492) and *CDK2* (cg00635560, cg16190688), were validated (*P* SMR < .05 & *P* SMR multi < .05 & *P* HEIDI > .05, Table 2), with the complete validation results presented in Table S3, Supplemental Digital Content, https://links.lww.com/MD/R241.

**Table 1:** UKB validation result: SMR analysis of mQTL–GWAS.

Probe ID	Gene symbol	*P* SMR	*P* SMR multi	*P* HEIDI	OR SMR (95% CI)
cg00635560	CDK2	8.69E-04	2.95E-03	6.61E-01	1.06 (1.02–1.1)
cg16190688	CDK2	2.00E-03	2.00E-03	7.90E-01	1.32 (1.11–1.58)
cg27365499	FBXO31	4.06E-02	4.06E-02	5.94E-01	0.82 (0.67–0.99)
cg27294008	NEK4	3.01E-02	3.63E-02	3.44E-01	1.08 (1.01–1.16)
cg10802521	NEK4	2.95E-02	1.36E-02	3.66E-01	0.95 (0.91–0.99)
cg09524078	NEK4	4.41E-02	4.41E-02	1.45E-01	1.27 (1.01–1.59)
cg24032190	SMAD3	1.56E-03	2.24E-02	2.28E-01	1.2 (1.07–1.34)

HEIDI = heterogeneity in dependent instrument, mQTL = methylation quantitative trait loci, SMR = summary-data-based Mendelian randomization, UKB = UK Biobank.

**Table 2:** GWAS catalog validation result: SMR analysis of mQTL–GWAS.

Probe ID	Gene symbol	*P* SMR	*P* SMR multi	*P* HEIDI	OR SMR (95% CI)
cg05715492	ARPC1B	1.56E-02	1.56E-02	8.23E-01	1.15 (1.03–1.28)
cg00635560	CDK2	1.60E-02	4.10E-02	5.31E-01	1.03 (1.01–1.06)
cg16190688	CDK2	3.15E-02	3.15E-02	7.04E-01	1.14 (1.01–1.29)
cg07800524	FGF21	1.87E-02	1.87E-02	7.50E-01	1.12 (1.02–1.23)
cg03531100	FGF21	2.57E-02	2.57E-02	7.02E-01	1.17 (1.02–1.34)
cg16155702	FGF21	2.08E-02	2.08E-02	7.92E-01	0.88 (0.79–0.98)
cg23514324	PPARG	3.53E-03	8.82E-03	7.42E-01	0.91 (0.86–0.97)
cg15585555	RASSF5	3.85E-03	3.36E-03	7.76E-01	0.82 (0.72–0.94)
cg03352169	SMAD3	6.98E-03	2.65E-02	2.76E-01	0.84 (0.74–0.95)
cg24032190	SMAD3	1.46E-03	1.06E-02	8.23E-01	1.14 (1.05–1.24)
cg13943355	SMAD3	3.78E-03	3.78E-03	5.36E-01	1.22 (1.07–1.39)
cg06469895	TERF2	3.76E-02	3.76E-02	8.90E-01	1.17 (1.01–1.37)
cg13518031	TP63	1.76E-02	1.76E-02	2.42E-01	1.21 (1.03–1.41)

HEIDI = heterogeneity in dependent instrument, mQTL = methylation quantitative trait loci, SMR = summary-data-based Mendelian randomization, UKB = UK Biobank.

### 3.2. Integration of RA and blood eQTL data related to cell senescence

**Figure 3.**
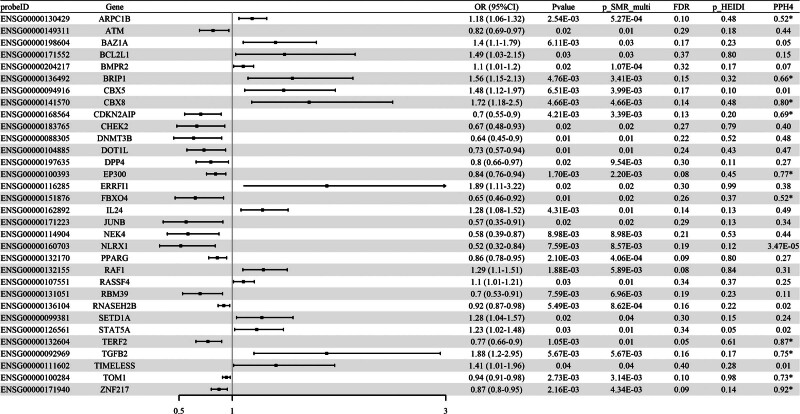
Association between cell senescence-related eQTL and RA–GWAS in SMR analysis (Forest map). *PPH4 > 0.5. CI = confidence interval; eQTL = expression quantitative trait loci; OR = odds ratio; RA = rheumatoid arthritis, SMR = summary-data-based Mendelian randomization.

SMR analysis was conducted to assess the association between cell senescence-related eQTL and RA–GWAS, with the complete analysis results presented in Table S4, Supplemental Digital Content, https://links.lww.com/MD/R241. We identified a total of 32 cell senescence-related genes associated with RA risk (*P* SMR < .05 & *P* SMR multi < .05 & *P* HEIDI > .05, Fig. 3). Among these, the expression levels of 15 genes, including *ARPC1B* (OR = 1.18, 95% CI [1.06–1.32]), *BAZ1A* (OR = 1.40, 95% CI [1.10–1.79]), *BCL2L1* (OR = 1.49, 95% CI [1.03–2.15]), *BMPR2* (OR = 1.10, 95% CI [1.01–1.20]), and *BRIP1* (OR = 1.56, 95% CI [1.15–2.13]), were positively correlated with RA risk. The remaining 17 genes, including *ATM* (OR = 0.82, 95% CI [0.69–0.97]), *CDKN2AIP* (OR = 0.70, 95% CI [0.55–0.90]), *CHEK2* (OR = 0.67, 95% CI [0.48–0.93]), *DNMT3B* (OR = 0.64, 95% CI [0.45–0.90]), and *DOT1L* (OR = 0.73, 95% CI [0.57–0.94]), were negatively correlated with RA risk. Co-localization analysis revealed strong evidence supporting the co-localization of 10 genes, including *ARPC1B* (PPH4 = 0.52), *BRIP1* (PPH4 = 0.66), *CBX8* (PPH4 = 0.80), *CDKN2AIP* (PPH4 = 0.69), and *EP300* (PPH4 = 0.77). However, none of the identified genes were validated in the UKB or GWAS Catalog cohorts (Tables S5 and S6, Supplemental Digital Content, https://links.lww.com/MD/R241).

### 3.3. Integration of RA and blood pQTL data related to cell senescence

**Figure 4.**
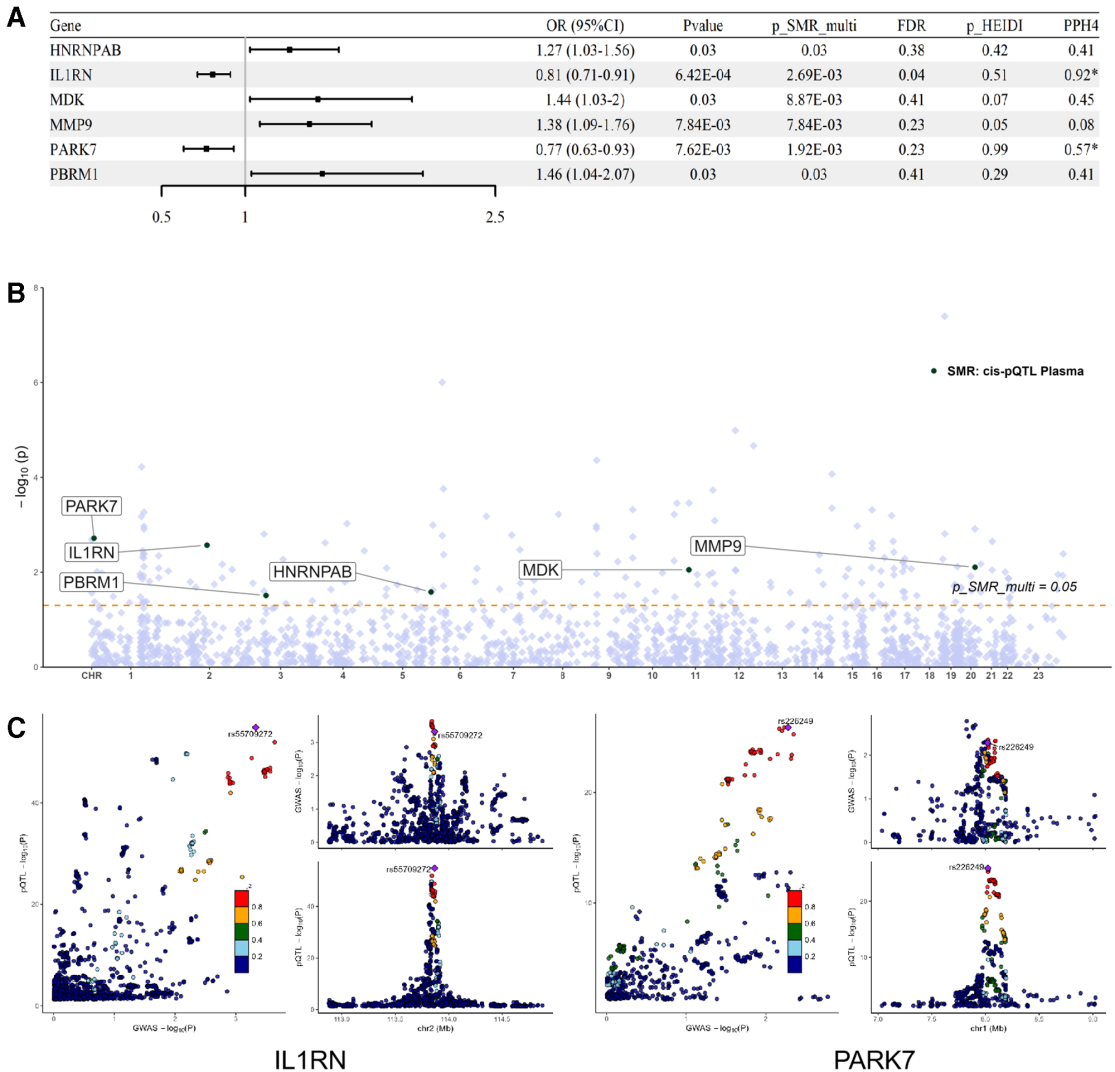
Association between cell senescence-related pQTL and RA–GWAS in SMR analysis. (A) SMR analysis results-Forest map. (B) Chromosome distribution of key proteins (Manhattan map). (C) Co-localization results. *PPH4 > 0.5. CI = confidence interval; OR = odds ratio; pQTL = protein quantitative trait loci; RA = rheumatoid arthritis, SMR = summary-data-based Mendelian randomization.

SMR analysis was conducted to assess the association between cell senescence-related pQTL and RA-GWAS, with results presented in Table S7, Supplemental Digital Content, https://links.lww.com/MD/R241. A total of 6 cell senescence-related proteins were identified as being associated with RA risk (*P* SMR < .05 & *P* SMR multi < .05 & *P* HEIDI > .05, Fig. 4A). Among them, the protein abundance of *HNRNPAB* (OR = 1.27, 95% CI [1.03–1.56]), *MDK* (OR = 1.44, 95% CI [1.03–2.00]), *MMP9* (OR = 1.38, 95% CI [1.09–1.76]), and *PBRM1* (OR = 1.46, 95% CI [1.04–2.07]) was positively correlated with RA risk. Conversely, the protein abundance of *IL1RN* (OR = 0.81, 95% CI [0.71–0.91]) and *PARK7* (OR = 0.77, 95% CI [0.63–0.93]) was negatively correlated with RA risk. Further analysis illustrated the chromosomal distribution of these proteins (Fig. 4B). Additionally, co-localization analysis revealed strong evidence supporting the co-localization of the proteins *IL1RN* (PPH4 = 0.92) and *PARK7* (PPH4 = 0.57), with co-localization results depicted in Figure 4C. These findings were not validated in the UKB or GWAS Catalog cohorts (Tables S8 and S9, Supplemental Digital Content, https://links.lww.com/MD/R241).

### 3.4. Integration of RA, mQTL, and eQTL data

Based on key results from SMR analyses of cell senescence-related blood mQTL and eQTL with RA-GWAS, we suggest that the genes *ERRFI1*, *PPARG*, *RAF1*, *NEK4*, *FBXO4*, *ARPC1B*, *ATM*, *RNASEH2B*, *BAZ1A*, *TERF2*, *STAT5A*, *CBX8*, *DOT1L*, *BCL2L1*, *DNMT3B*, and *TOM1* may have a causal association with RA. Subsequently, we conducted an SMR analysis using mQTL as the exposure and eQTL as the outcome variables to explore whether methylation of CpG sites in these overlapping results significantly regulates the expression of the corresponding genes. The full results of this analysis are provided in Table S10, Supplemental Digital Content, https://links.lww.com/MD/R241). Further analysis based on these results revealed significant validation for *BCL2L1* (cg12873919, cg13989999), *DNMT3B* (cg09149842, cg13788819, cg21235334, cg26553763), *ERRFI1* (cg13808198, cg22678073), *NEK4* (cg09524078), and *RAF1* (cg26432171) (*P* SMR < .05 & *P* SMR multi < .05 & *P* HEIDI > .05, Table 3). Similarly, considering the key findings from the eQTL–pQTL integration analysis alongside the SMR analysis of pQTL and RA–GWAS, no positive overlapping results were found; therefore, no further SMR analysis was performed integrating eQTL–pQTL data.

**Table 3:** Integrated SMR analysis results of mQTL–eQTL.

Expo ID	Outco gene	*P* SMR	*P* SMR multi	*P* HEIDI	OR SMR (95% CI)
cg12873919	BCL2L1	1.40E-07	1.40E-07	1.97E-01	0.82 (0.76–0.88)
cg13989999	BCL2L1	1.43E-06	1.43E-06	2.36E-01	0.78 (0.71–0.87)
cg09149842	DNMT3B	1.22E-09	1.22E-09	5.41E-02	1.21 (1.14–1.29)
cg13788819	DNMT3B	2.64E-10	2.64E-10	2.67E-01	1.18 (1.12–1.25)
cg21235334	DNMT3B	8.34E-10	8.34E-10	5.06E-02	1.2 (1.13–1.27)
cg26553763	DNMT3B	3.14E-07	3.14E-07	5.48E-02	0.77 (0.69–0.85)
cg13808198	ERRFI1	2.45E-05	2.45E-05	8.09E-01	0.77 (0.68–0.87)
cg22678073	ERRFI1	1.86E-09	5.02E-07	2.67E-01	0.94 (0.92–0.96)
cg09524078	NEK4	1.87E-05	1.87E-05	6.00E-02	0.78 (0.69–0.87)
cg26432171	RAF1	9.18E-09	9.18E-09	5.97E-02	1.83 (1.49–2.25)

eQTL = expression quantitative trait loci, HEIDI = heterogeneity in dependent instrument, mQTL = methylation quantitative trait loci, SMR = summary-data-based Mendelian randomization.

### 3.5. Multi-omics data integration analysis

**Figure 5.**
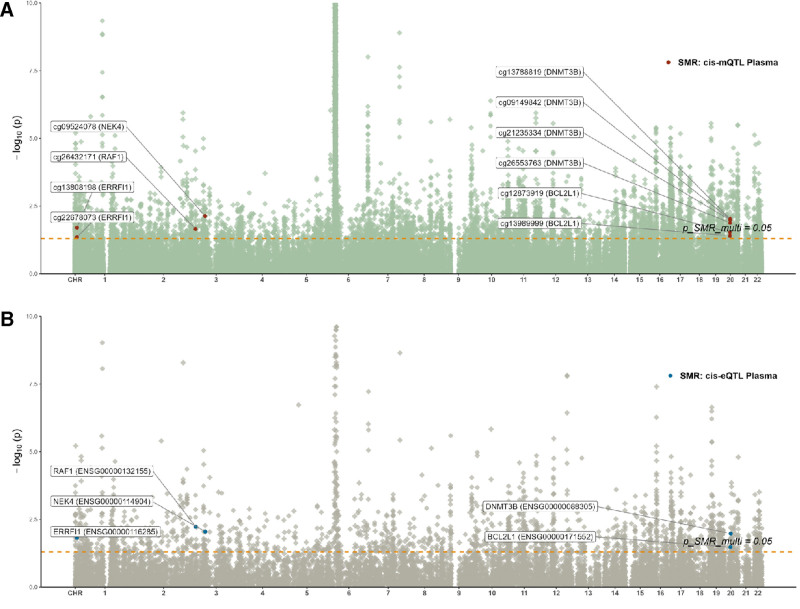
Chromosome distribution of key gene methylation and expression in SMR analysis (Manhattan map). (A) Gene methylation. (B) Gene expression. SMR = summary-data-based Mendelian randomization.

Upon integrating evidence across multiple omics levels, we identified that *BCL2L1* (cg12873919, cg13989999), *DNMT3B* (cg09149842, cg13788819, cg21235334, cg26553763), *ERRFI1* (cg13808198, cg22678073), *NEK4* (cg09524078), and *RAF1* (cg26432171) may have causal associations with RA. The chromosomal distribution of these key loci and their corresponding genes is shown in Figure 5. Subsequent analyses revealed that these loci and their associated genes showed significant validation in the SMR analysis of mQTL, eQTL, and RA–GWAS, as well as in the integrated mQTL–eQTL analysis. Specifically, the methylation site cg09524078 in *NEK4* was strongly supported in the co-localization analysis between mQTL and RA–GWAS (PPH4 > 0.5), while the remaining loci and genes did not receive strong evidence in co-localization analysis between mQTL, eQTL, and RA–GWAS (PPH4 < 0.5). By evaluating the odds ratio (OR) values, we determined the risk correlation and regulatory direction. Based on SMR analysis, the mQTL effect sizes at sites such as cg13989999, cg12873919, cg13808198, and cg22678073 are negatively associated with RA risk, suggesting that genetically regulated reductions in methylation may be related to an increased risk of the disease. The expression levels of *BCL2L1* and *ERRFI1* showed positive associations with RA risk, while the expression level of *NEK4* was negatively associated with RA risk. Genetic regulation of methylation variation at loci cg13989999, cg12873919, cg13808198, cg22678073, and cg09524078 was inversely correlated with the expression levels of their corresponding genes. Similarly, mQTL effects at loci cg21235334, cg13788819, and cg09149842 demonstrated negative associations with RA risk, whereas loci cg26553763 and cg26432171 showed positive associations. The expression level of *DNMT3B* was negatively associated with RA risk, while *RAF1* expression exhibited a positive association. The mQTL signals at loci cg21235334, cg13788819, cg09149842, and cg26432171 were positively linked to the expression of their respective genes, whereas methylation at locus cg26553763 showed an inverse relationship. In summary, the SMR-derived genetically regulated methylation variations suggest that reduced methylation at loci cg13989999 and cg12873919 may upregulate *BCL2L1* expression, while similar effects at cg13808198 and cg22678073 may enhance *ERRFI1* expression. Conversely, elevated mQTL effects at cg26432171 were associated with increased *RAF1* expression. These regulatory patterns align with heightened RA risk. Likewise, reduced mQTL signals at loci cg21235334, cg13788819, and cg09149842 or elevated signals at cg26553763 may suppress *DNMT3B* expression, whereas increased methylation effects at cg09524078 may downregulate *NEK4* expression, collectively contributing to RA susceptibility.

**Figure 6.**
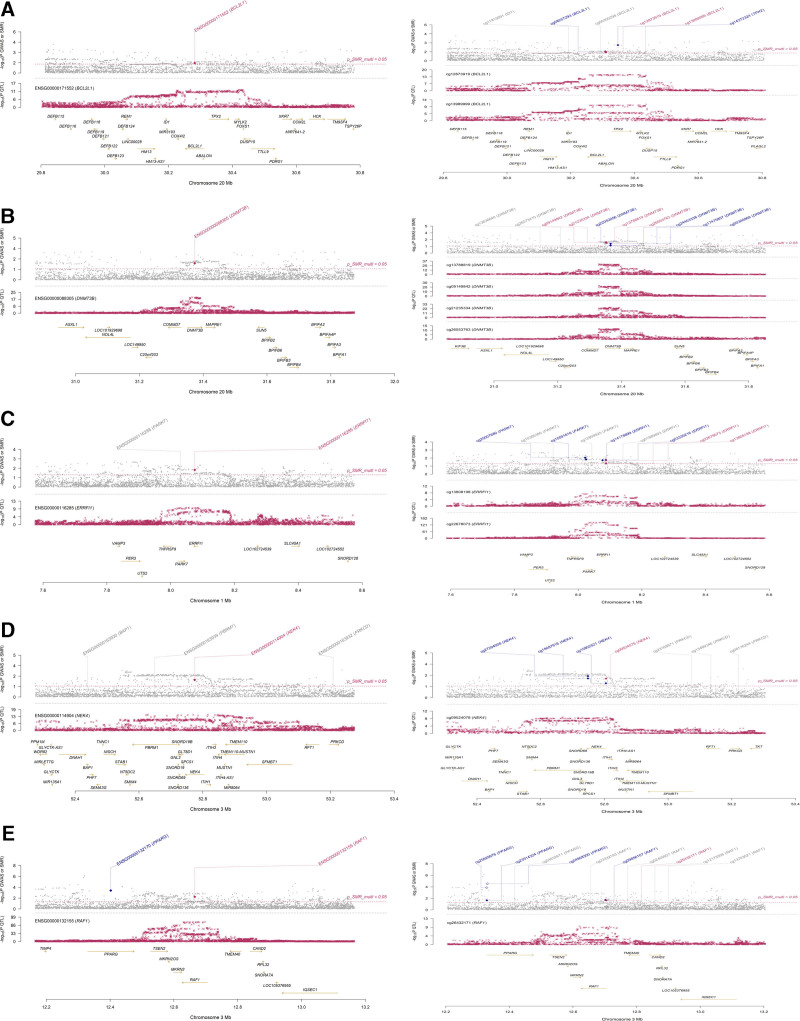
SMR locus plot results. (A) BCL2L1 (cg12873919, cg13989999); (B) DNMT3B (cg09149842, cg13788819, cg21235334, cg26553763); (C) ERRFI1 (cg13808198, cg22678073); (D) NEK4 (cg09524078); (E) RAF1 (cg26432171). The target genes are marked in red, with a threshold of *P* SMR multi = .05, and genes showing significance within the window region are indicated in blue, while the remaining genes are shown in gray. Genes that passed the HEIDI test (*P* HEIDI > .05) are marked with ◆, and those that did not pass are marked with ◇. HEIDI = heterogeneity in dependent instrument, SMR = summary-data-based Mendelian randomization.

**Figure 7.**
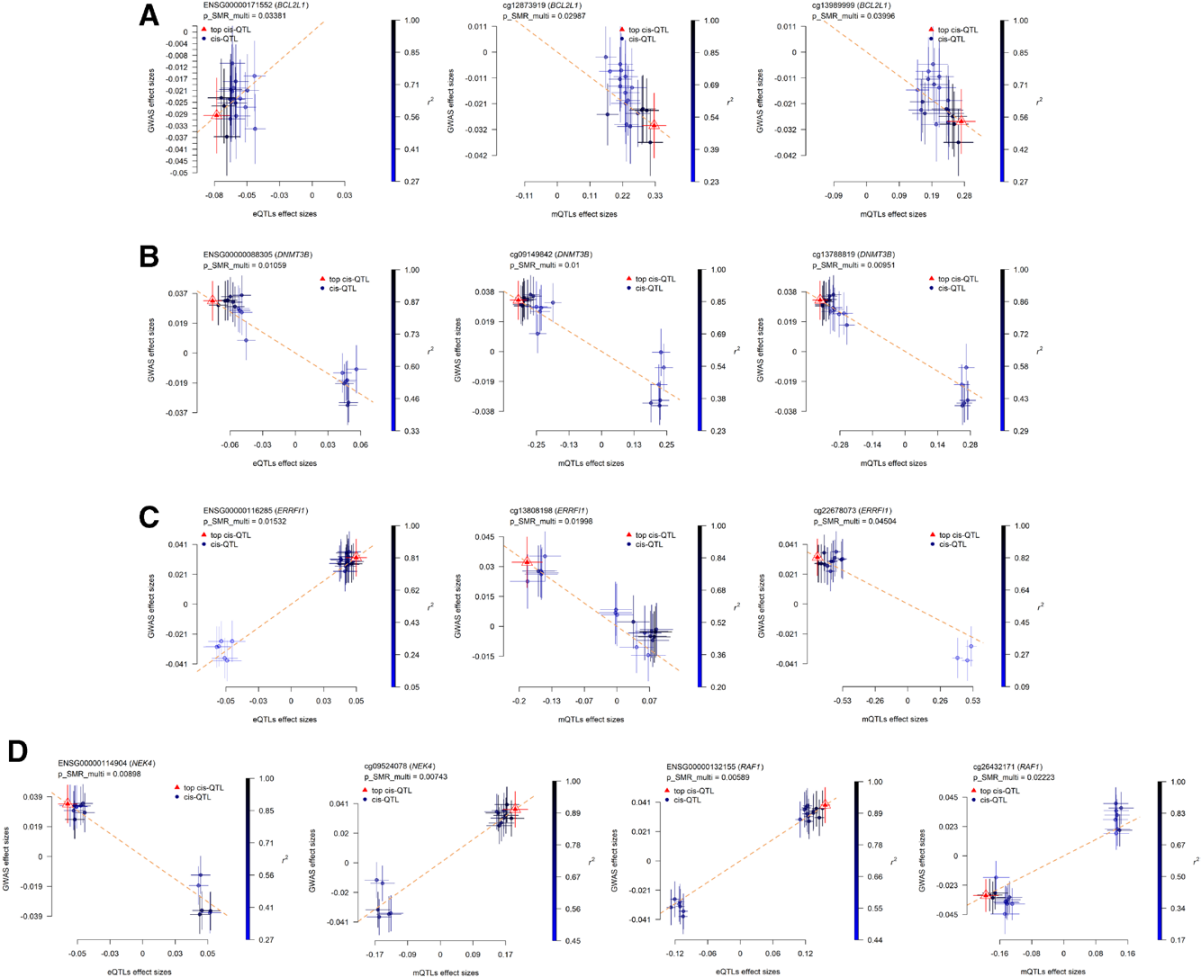
SMR EffectPlot results. (A) BCL2L1 (cg12873919, cg13989999); (B) DNMT3B (cg09149842, cg13788819, cg21235334, cg26553763); (C) ERRFI1 (cg13808198, cg22678073); (D) NEK4 (cg09524078); (E) RAF1 (cg26432171). SMR = summary-data-based Mendelian randomization.

Additionally, we utilized SMR LocusPlot analysis to demonstrate the genetic concordance within a 500 kb window for mQTL, eQTL, and RA–GWAS of *BCL2L1* (cg12873919, cg13989999), *DNMT3B* (cg09149842, cg13788819, cg21235334, cg26553763), *ERRFI1* (cg13808198, cg22678073), *NEK4* (cg09524078), and *RAF1* (cg26432171) (*P* SMR multi < .05, Fig. 6). Certain SNPs were found to be associated with the regulation of candidate gene expression and with genetic susceptibility to disease, suggesting that the expression or methylation of these genes may play a crucial role in the pathogenesis of RA. Further SMR EffectPlot analysis indicated a significant correlation between the methylation or gene expression levels at specific loci and GWAS effect sizes, highlighting a potential causal relationship between methylation, gene expression, and disease risk (*P* SMR multi < .05, Fig. 7). Through SMR EffectPlot analysis, we further confirmed the link between methylation/gene expression and RA susceptibility, supporting their functional roles in the disease.

## 4. Discussion

In this study, we systematically evaluated the associations between cell senescence-related mQTL, eQTL, pQTL, and RA risk by integrating multi-omics data and employing SMR and co-localization analyses. The results indicate that cell senescence-related genes *BCL2L1*, *DNMT3B*, *ERRFI1*, *NEK4*, and *RAF1* are significantly associated with RA risk, supported by multi-omics evidence, highlighting the importance of these genes in the pathology of RA.

*BCL2L1* encodes the *BCL-xL* protein, which primarily functions to inhibit apoptosis, particularly under stress conditions, by preventing the action of pro-apoptotic proteins such as *BAX*, thereby maintaining cell survival.^[17]^ During cell senescence, the expression of *BCL-xL* typically increases, which is associated with enhanced resistance to apoptosis.^[18,19]^ This enhanced anti-apoptotic capability is especially significant in RA, as it may lead to the prolonged survival of immune cells, thereby exacerbating chronic inflammatory responses. In RA patients, the over-expression of *BCL-xL* may increase the survival of pathological cells, particularly within the inflammatory microenvironment, potentially resulting in further joint tissue damage and the spread of inflammation.^[20]^ Studies have also shown that *BCL-xL* promotes the transition of cells into a pre-senescent state during the senescence process and reduces sensitivity to genotoxic stress, thereby preventing cells from entering the apoptotic pathway, a phenomenon particularly evident in elderly RA patients.^[21]^ Additionally, *BCL-xL* can influence the cell senescence process by regulating autophagy, an important protective mechanism for cells under stress.^[22,23]^ Our SMR analysis results indicate a significant causal association between the *BCL2L1* gene and RA risk. Specifically, low mQTL effects at the cg13989999 and cg12873919 loci of the *BCL2L1* gene can enhance *BCL2L1* expression, thereby increasing RA risk. Thus, *BCL2L1* may influence RA pathogenesis by regulating cell senescence mechanisms and apoptosis functions. DNA methylation plays a crucial role in gene expression regulation and chromatin structure, and its proper establishment and maintenance are vital for mammalian development and cell differentiation. *DNMT3B* is a major DNA methyltransferase involved in regulating DNA methylation, thereby influencing gene expression patterns and cell fate.^[24]^ During cell senescence, abnormal *DNMT3B* function may lead to alterations in DNA methylation patterns, which are closely associated with aging-related diseases, including RA. Specifically, in RA patients, *DNMT3B* may exacerbate immune overreaction and joint inflammation by aberrantly regulating the expression of genes related to immune response and inflammation through abnormal methylation.^[25,26]^ Our study found that the expression level of the *DNMT3B* gene is negatively correlated with RA risk, with low mQTL effects at its key loci, cg21235334, cg13788819, and cg09149842, inhibiting *DNMT3B* expression, thereby regulating the corresponding signaling pathways. It is well known that the *EGFR* signaling pathway plays a crucial role in RA pathogenesis. In our SMR analysis, we also found a significant causal association between *ERRFI1* and RA. The protein encoded by the *ERRFI1* gene is a negative regulator of cell proliferation and apoptosis, functioning by inhibiting the *EGFR* signaling pathway.^[27]^ In our single-cell sequencing study (unpublished data), *ERRFI1* was found to be highly expressed in the synovial tissue of RA patients, predominantly in FLS. We speculate that excessive *ERRFI1* activity may lead to uncontrolled proliferation of FLS and joint tissue destruction, thereby inducing sustained chronic inflammation, although the specific regulatory mechanisms require further research. Research has also identified that the *NEK4* gene plays an important role in cell cycle regulation and DNA damage response. Abnormal *NEK4* expression is closely related to cell senescence, particularly in response to DNA damage, where its dysfunction may lead to cell cycle arrest or incomplete repair, thereby promoting cell senescence.^[28]^ As an important regulator of cell senescence and genomic stability, *NEK4* holds potential significance in understanding diseases related to DNA damage and cell senescence. In addition to the aforementioned genes, variations in *RAF1* and its loci are also factors that increase RA risk. The *RAF1* gene plays a key role in the MAPK/ERK signaling pathway, which regulates cell proliferation, differentiation, and survival.^[29,30]^ Abnormal activation of *RAF1* is associated with enhanced cell senescence and inflammatory responses, which are particularly evident in RA patients. In some cases, excessive activation of *RAF1* can lead to uncontrolled cell proliferation and may induce premature senescence, accompanied by heightened stress responses and inflammatory signaling. On the other hand, *RAF1* also regulates the cell senescence process by influencing telomere maintenance and the DNA damage response.^[31,32]^ *RAF1* dysfunction may be linked to reduced DNA damage repair capacity, thereby promoting cell senescence and genomic instability, which are closely associated with the pathogenesis of various aging-related diseases. Studies have indicated that abnormal expression of *RAF1* in the synovial tissue of RA patients may promote cell migration, invasion, and inflammatory responses, accelerating disease progression.^[33]^ Our findings underscore the critical role of *RAF1* in RA pathogenesis and suggest that it may be a potential therapeutic target for RA.

A significant strength of this study lies in the combined use of SMR and co-localization analysis, which systematically assesses the causal relationships between methylation, expression, and protein abundance of cell senescence genes and RA risk by leveraging genetic variation. The SMR analysis effectively integrates data from different omics layers, thereby enhancing the credibility of the causal associations between genes and disease risk. The consistency of results across multiple independent datasets further supports the crucial role of cell senescence-related genes in the pathogenesis of RA. Despite revealing the potential associations between cell senescence-related genes and RA through multi-omics integration, this study has several limitations. First, the QTL data primarily originate from blood or peripheral tissues of European ancestry populations (such as eQTLGen and mQTL cohorts), whereas the core pathological mechanisms of RA may involve synovial tissue-specific regulation. For instance, RA–FLSs are key effector cells driving RA progression, and their senescent phenotypes and regulatory networks (such as specific epigenetic marks or non-coding RNA regulation) may differ significantly from the senescence signals observed in peripheral blood cells. Therefore, whether the signals identified in the blood in the current study can directly reflect the pathological mechanisms in the synovial tissue still requires specific validation in RA synovial tissue in future work. Second, although MR analysis can reduce confounding bias, the selection of instrumental variables relies on the statistical power of existing GWAS and QTL data, potentially overlooking rare variants or sex/age-specific effects. Subsequent studies could enhance causal inference by including multi-ethnic cohorts (such as East Asian GWAS) and longitudinal QTL data across multiple time points. Another limitation of this study is the lack of independent external validation for our identified eQTL–RA and pQTL–RA associations. This is primarily for 2 reasons: 1st, large-scale eQTL and pQTL datasets that are publicly available, match the tissue of our discovery cohort (whole blood), and possess sufficient statistical power remain scarce; second, as reported in the literature, the heritability of gene expression and protein levels is often lower than that of epigenetic marks like DNA methylation, making the robust replication of these associations across different cohorts inherently challenging. Therefore, these eQTL and pQTL findings should be regarded as exploratory, supportive evidence for the mQTL co-localization signals, and their precise causal relationships await confirmation in future, larger-scale studies. Regarding sensitivity analysis, we did not perform additional analyses (e.g., IVW-MR or MR-Egger) on the eQTL/pQTL–RA SMR results. We assessed the feasibility of such analyses, but considered that the primary purpose of the eQTL/pQTL analyses in this study was to serve as multi-omic supportive evidence for the mQTL–RA signals, rather than as independent causal inference. Given that these signals were relatively weak and potentially susceptible to weak instrument bias, we concluded that introducing these additional MR analyses would likely not provide substantial further support for the core conclusions of our study (which are primarily based on the mQTL–RA co-localization analysis). Consequently, we opted to discuss the lack of validation transparently as a limitation, rather than introducing additional analyses that might be constrained by their own methodological assumptions. Our current research focuses mainly on GWAS results and SNP association analysis, but the functional exploration of genes remains limited. Future studies could integrate more advanced genomics to further investigate the specific mechanisms of candidate genes. Additionally, the SMR method cannot provide the absolute methylation differences of CpG sites as in traditional case–control studies. Its results reflect the potential associations between genetically regulated methylation variations and phenotypes rather than direct measurements.

In conclusion, this study explores the potential causal relationships between the methylation, expression, and protein abundance of cell senescence-related genes and RA through SMR and co-localization analyses. Multi-omics studies have also validated the possible key role of cell senescence-related genes in the pathogenesis of RA. Future research should further investigate the specific functional mechanisms of these genes and evaluate their potential clinical application value in RA patients. With the advancement of high-throughput omics technologies, integrating multi-omics data will become a crucial direction in uncovering the complex pathological mechanisms of RA.

## Author contributions

**Data curation:** Youji Jia.

**Formal analysis:** Yajuan Guo.

**Investigation:** Ping Jiang.

**Methodology:** Zhi Wang.

**Software:** Honghong Ma, Mingcong Wang.

**Supervision:** Wei Yan, Xiaobing Xi.

**Writing – original draft:** Ping Jiang.

**Writing – review & editing:** Juhua Zhang, Xiaobing Xi.

## Supplementary Material


